# Association Between the Two-Year Trajectories of Dental Anxiety and the Changes in the Oral Health-Related Quality of Life in Parents of FinnBrain Birth Cohort Study

**DOI:** 10.3390/dj12120398

**Published:** 2024-12-06

**Authors:** Lily Yang, Auli Suominen, Katri Palo, Eeva-Leena Kataja, Vesa Pohjola, Mika Ogawa, Linnea Karlsson, Hasse Karlsson, Eero Laakkonen, Satu Lahti

**Affiliations:** 1 Department of Community Dentistry, University of Turku, 20014 Turku, Finland; lily.a.yang@utu.fi (L.Y.); katri.palo@utu.fi (K.P.); vesa.pohjola@utu.fi (V.P.); mika.ogawa@utu.fi (M.O.); 2 FinnBrain Birth Cohort Study, Department of Clinical Medicine, Turku Brain and Mind Center, University of Turku, 20014 Turku, Finland; eeva-leena.kataja@utu.fi (E.-L.K.); linnea.karlsson@utu.fi (L.K.); hasse.karlsson@utu.fi (H.K.); 3 Centre for Population Health Research, University of Turku, 20014 Turku, Finland; 4 Research Unit of Population Health, Faculty of Medicine, University of Oulu, 90570 Oulu, Finland; 5 Department of Public Health, Turku University Hospital, University of Turku, 20520 Turku, Finland; 6 Department of Child Psychiatry, Turku University Hospital, 20520 Turku, Finland; 7 Department of Psychiatry, Turku University Hospital, University of Turku, 20520 Turku, Finland; 8 Department of Teacher Education, University of Turku, 20014 Turku, Finland; eero.laakkonen@utu.fi

**Keywords:** dental fear, anxiety, oral health-related quality of life

## Abstract

**Objectives**: We evaluated associations between changes in dental anxiety and oral health-related quality of life (OHRQoL) in parents of the FinnBrain Birth Cohort Study. **Methods**: Two-year dental anxiety trajectories measured with Modified Dental Anxiety Scale from gestational weeks (gw) 14 and 34, and 3 and 24 months after birth were used. OHRQoL was measured with the Oral Health Impact Profile 14-item questionnaire at gw34 and 4 years. Changes in the OHRQoL sum and dimension scores according to dental anxiety trajectories were analyzed with the Jonckheere–Terpstra test separately for mothers (*n* = 998) and fathers (*n* = 513). **Results**: Overall, OHRQoL decreased in all dental anxiety trajectory groups except the High decreasing group in mothers and in fathers. The decline in the overall OHRQoL was greatest in the Stable high trajectory group for both parents. In fathers, OHRQoL increased considerably but not statistically significantly in the High decreasing trajectory group. The changes in OHRQoL dimensions Psychological discomfort and Handicap differed according to dental anxiety trajectories for both of mothers and fathers, and also for the dimension Psychological disability for mothers. **Conclusions**: These findings highlight the need for targeted interventions in treating high dental anxiety and in preventing the increase in dental anxiety to improve oral health outcomes such as OHRQoL.

## 1. Introduction

The WHO Quality of Life (QoL) group has defined QoL as “individuals’ perception of their position in life in the context of the culture and value systems in which they live and in relation to their goals, expectations, standards and concerns” [1]. It has been included into health research and services to incorporate important patient perspective. Oral health-related quality of life (OHRQoL) is a multidimensional construct that evaluates patients’ oral health, functional well-being, emotional well-being, expectations and satisfaction with care and sense of coherence [2,3]. Those with common oral health problems such as caries, periodontal disease and missing teeth report poorer OHRQoL [4,5,6,7]. Dental anxiety is another common oral health-related problem [8,9] which leads to irregular attendance [10]. Patients with high dental anxiety and irregular dental attendance have a four-times-higher risk of poor OHRQoL in comparison with those who have lower levels of dental anxiety or who attend dentist regularly [11]. Additionally, post childhood irregular dental visiting patterns have been associated with lower OHRQoL in adulthood [12].

Numerous studies consistently reported that higher dental anxiety has been related to poorer OHRQoL among adults and adolescents [13,14,15,16,17,18,19]. Differences between those with high dental anxiety and low dental anxiety have been found especially in psychological, social and handicap dimensions of OHRQoL showing that those with high dental anxiety experienced embarrassment and dissatisfaction with life in general more often [14].

According to our knowledge, so far, there are few longitudinal studies on the association between changes in dental anxiety and OHRQoL. A small study of 35 patients with high dental anxiety showed that a reduction in dental anxiety was associated with an improvement in OHRQoL, and that reduction in dental anxiety, rather than improved oral health, predicted an improved OHRQoL [20]. This suggests that decreasing dental anxiety might have a direct positive effect on OHRQoL. However, the association between changes in dental anxiety and OHRQoL needs to be confirmed in larger samples at the population level.

Thus, the aim of this study was to analyze whether the trajectories of changes in dental anxiety are associated with the changes in the OHRQoL and its dimensions in the parents of the FinnBrain Cohort Study. We hypothesize that stable high dental anxiety is associated with stable poor OHRQoL, and decreased dental anxiety with the improvement of OHRQoL.

## 2. Materials and Methods

This longitudinal study is a secondary analysis of the longitudinal data from the FinnBrain Birth Cohort Study (http://www.finnbrain.fi/), which prospectively studies the effects of environment and genes on children’s brain development and health [21]. The participants of the study were recruited after ultrasonography appointments that are offered free of charge to every pregnant woman in Finland by municipal maternity clinics during the first trimester of the pregnancy. Expecting mothers and their partners who participated the appointments in the South-Western Hospital District and the Åland Islands in Finland in 2011–2015 were invited to participate the study. Mothers were asked to invite those partners (later called fathers) who did not participate the appointment. The Ethics Committee of the Hospital District of Southwest Finland has approved the study protocol (14.6.2011 ETMK: 57/180/2011 § 168). Written informed consent was obtained from all participants.

Of the 5790 pregnant mothers visiting the recruitment site and invited to the study, 3808 mothers and 2623 fathers expecting decided to participate [21]. The parents selected the mode of questionnaire they preferred. The data for this study were collected by paper or electronic questionnaires between gestational week (gw) 14 and at 4 years after childbirth. The trajectory solution for dental anxiety between gw14 and 24 months after childbirth was obtained for 3201 mothers and 2068 fathers. Of those with the trajectory solution, 1039 mothers (27% of participants) and 513 fathers (20% of participants) returned the OHRQoL questionnaires at both time points, gw34 and 4 years. Regarding the mothers, 41 did not meet the inclusion criteria (did not answer the questions concerning OHRQoL). Thus, the total numbers included in the analysis was 998 mothers and 513 fathers. To justify the use of the same trajectory groups, we ensured that this sample population was similar enough with the original population for which the trajectories were calculated. We calculated means, standard deviations and 95%CIs of the Modified Dental Anxiety Scale (MDAS) scores from non-imputed data in each time point and according to the trajectory groups for those with information available at all measurement points (mean, SD) and for those with information available at least in one measurement point (95%CI). Parents’ background information on age and education were collected during pregnancy. In this study, educational level was divided into three levels: low (compulsory, vocational or secondary level, 12 years of schooling), medium (polytechnics) and high (university degree).

Dental anxiety was measured with validated Finnish version of MDAS which consists of five questions for self-rating dental anxiety [22,23,24,25]. The questions in the MDAS were as follows: (1) If you went to your dentist for treatment tomorrow, how would you feel? (2) If you were sitting in the waiting room (waiting for treatment), how would you feel? (3) If you were about to have a tooth drilled, how would you feel? (4) If you were about to have your teeth scaled and polished, how would you feel? (5) If you were about to have a local anesthetic injection in your gum, above an upper back tooth, how would you feel? Each question was rated on a five-point scale ranging from 1 (not anxious) to 5 (extremely anxious); summated scores ranged from 5 to 25. The MDAS was measured on following four points: at gestational weeks (gw) 14 and 34, and 3 and 24 months after birth. Dental anxiety trajectories (four for mothers and five for fathers) that were identified using latent growth mixture modeling and reported in a previous study from the same population were used [26].

The OHRQoL was measured by the validated Finnish translation of Oral Health Impact Profile 14 (OHIP-14) questionnaire at gw34 and 4 years [27,28]. It contains 14 questions about the frequency of adverse impacts during the preceding month of the following five-point scale ranging from 0 (never) to 4 (very often). The severity sum score was calculated as sum of the ordinal responses. Lower scores indicated a better OHRQoL. The severity sum change score was calculated by extracting the 4-year score from gw34 score, negative scores indicating deteriorating OHRQoL. Correspondingly, sum and change scores were calculated for the seven dimensions of OHRQoL, that is, Functional limitation, Physical pain, Psychological discomfort, Physical disability, Psychological disability, Social disability and Handicap.

The cross-tabulations and descriptive statistics were used for examination of age, education, dental anxiety trajectories, severity and dimension scores for mothers and fathers. The OHIP-14 severity scores at gw34 and 4 years were compared in each trajectory group with Wilcoxon rank sum test. The association between dental anxiety trajectories and change between gw34 and 4 years in the OHIP-14 severity scores by total and dimensions was analyzed with the Jonckheere–Terpstra test separately for mothers and fathers using the ascending order based on the dental anxiety level at 24 months. The corresponding effect sized were calculated using the matched-pairs rank biserial correlation coefficient. The statistical analyses were processed with SPSS Statistics for Windows (Version 29.0., IBM Corp: Armonk, NY, USA). The *p*-values of <0.05 were considered to be indicative of the statistical differences.

## 3. Results

The comparison showed sufficient similarity between the groups (Table 1). There were no systematic differences in the mean dental anxiety levels between the original sample used to calculate dental anxiety trajectories and the sample used in this study as the 95% confidence intervals overlapped largely.

Table 2 presents the distribution of the participants according to their OHRQoL, age and education. Both mothers and fathers experienced an increase in the severity of OHIP-14 scores over the 4-year period, indicating a decline in OHRQoL. Mothers experienced approximately 0.5 points greater deterioration in OHRQoL than fathers during the study period. The OHRQoL tended to decrease according to most OHIP-14 dimensions for both mothers and fathers. The change was greatest in Physical Pain and Psychological Discomfort dimensions.

Table 3 shows the OHRQoL (OHIP-14 severity) at two time points (gw34 and 4-year follow-up) and the change in OHRQoL according to different dental anxiety trajectories among mothers and fathers. Negative change in OHIP-14 severity scores indicated a slight but statistically significant decrease in OHRQoL, in all except one trajectory group in mothers and in two groups in fathers. The OHRQoL differed according to dental anxiety trajectories in mothers at both time points and in fathers at 4 years. The difference in the OHRQoL change between dental anxiety trajectory groups was statistically significant in mothers and fathers. OHRQoL decreased most in mothers and fathers in the Stable high trajectory groups. In mothers, OHRQoL decreased second most in the Moderate increasing trajectory group. In fathers, there was a considerable increase in OHRQoL in the High decreasing trajectory group, but the difference was not statistically significant. In mothers, the effect sizes were medium, except for the Stable low group for which it was small. In fathers, the effect size varied more. For the Stable high group, the effect size was large; for the Moderate increasing group, medium; and for other groups, small or not calculatable.

Table 4 presents the change in the scores of the seven dimensions of OHIP-14 according to dental anxiety trajectories. Of the dimensions, the changes in Psychological discomfort and Handicap differed according to dental anxiety trajectories in both of mothers and fathers, and in Psychological disability in mothers. In mothers, the decrease in OHRQoL (negative change score) was the greatest in the Stable high trajectory groups for Psychological disability and Handicap dimensions, and for Psychological discomfort, the decrease was highest in the Moderate increasing trajectory group and second highest in the Stable high trajectory group. In fathers, the decrease in the Psychological discomfort and Handicap dimensions of OHRQoL were highest in the Stable high trajectory group, but increase was observed in the High decreasing group.

## 4. Discussion

The OHRQoL decreased most in the Stable high dental anxiety trajectory groups in both parents. The changes in the OHRQoL dimensions Psychological disability and Handicap differed according to dental anxiety trajectories for both parents, the decrease being greatest in the Stable high trajectory groups.

A previous longitudinal study [20] demonstrated that reductions in dental anxiety led to improvements in OHRQoL, whereas improvements in oral health (rated by dentists or patients) did not show a consistent association with OHRQoL improvement. Other studies reporting changes in dental anxiety over several time points [26,29] did not include OHRQoL, and the studies with changes in OHRQoL did not include dental anxiety. In a Finnish nationally representative longitudinal survey, the mean OHIP-14 severity improved from 2000 to 2011 from 2.90 to 2.41 (*p* = 0.197) in men and from 2.41 to 1.98 (*p* = 0.004) in women of similar age with this age group [30], while in this study, the OHRQoL declined. In general, after treatment of the most common oral problems caries and periodontal disease, the improvement in OHRQoL has been moderate [31,32], but not statistically significant [33].

In most of the cross-sectional studies, those with high dental anxiety have reported a lower OHRQoL than those with low dental anxiety [13,14,15,16,17,18,19] even in a nationally representative population when adjusted for age, gender, education, remaining teeth, and dental visits [14]. These findings from cross-sectional studies align with our present findings, where those in the Stable low dental anxiety group reported a better OHRQoL at both time points than those in other trajectory groups. On the other hand, the OHRQoL in our study was better even in the Stable high anxiety group than in highly dentally anxious Dutch patients (4.0 in mothers and 7.6 in fathers and 30.5 in Dutch patients) [34]. In a previous study among adults in Finland, the greatest differences between those with high and low dental anxiety were found in psychological, social, and handicap dimensions of OHIP-14 [14]. These findings are in concordance with those of the present study, where the greatest decrease in the OHRQoL was seen in Psychological Disability and Handicap dimensions. Dental anxiety has tended to show a strong effect on psychosocial impact measured with the Handicap dimension [35], but an even greater effect on the Psychological discomfort, Psychological disability, Physical pain, Social disability and Physical disability dimensions in those with high dental anxiety [34]. The improvements in these dimensions were also greater than in the Handicap dimensions in another study [35].

The large sample size and rather long follow-up periods in addition to the use of valid and reliable methods were the strengths of this study. On the other hand, different follow-up periods for the dental anxiety trajectories and changes in the OHRQoL can be considered as a limitation. However, dental anxiety had been shown to be rather stable in adulthood over 11 years [8], and thus, those with stable high dental anxiety especially could be likely to remain so until the 4-year measurement point. The use of the trajectory solution obtained from a larger sample can be consider as a limitation as this needs to be applied with a caution [36,37]. As the High decreasing group among fathers was already small in the largest sample, this led to very small number of fathers in the decreasing high dental anxiety trajectory group in this study population [26]. However, the MDAS scores in this study and the original study population were rather similar even though those staying in the study over four years were more often older and had higher education. As this group was originally selected partly based on clinical relevance, the current finding of an improvement in their OHRQoL looks promising but needs to be confirmed in another larger sample.

## 5. Conclusions

The findings of this study emphasize the impact of dental anxiety and its changes on changes in OHRQoL over time among mothers and fathers. They also suggest gender differences in these changes. It also highlights the need for targeted interventions both in treating high dental anxiety and in preventing the increase in dental anxiety to improve oral health outcomes. The interventions include efficient basic dental anxiety management techniques, such as building trust, providing control (such as cognitive or behavioral), using a tell–show–do method and relaxation strategies (such as muscle or breathing relaxation) [38], are useful to reduce and prevent the development of dental anxiety. Further research is needed in other populations and on the effects of these dental anxiety management interventions on changes in OHRQoL.

## Figures and Tables

**Table 1:** Dental anxiety levels (mean, standard deviation, 95% CI) for the original dental anxiety trajectories data and for data from the sample included in this study (non-imputed values). Number and percentages for those with information available at all measurement points ^a^ and for those with information available at least in one measurement point ^b^.

Trajectories	Total *n* (%)				Gestational Week 14		Gestational Week 34		3 Months		2 Years	
	Original		Sample		Original	Sample	Original	Sample	Original	Sample	Original	Sample
Mothers	*n* = 3201		*n* = 998									
Stable low	960 (30.0) ^a^	2583 (80.7) ^b^	826 (82.8)	mean (SD)	8.9 (2.8)	8.7 (2.7)	8.7 (2.7)	8.6 (2.6)	9.1 (2.9)	8.9 (2.7)	9.1 (2.9)	9.0 (2.8)
				95% CI	8.8–9.0	8.5–8.9	8.6–8.8	8.4–8.8	9.0–9.2	8.7–9.1	8.9–9.3	8.8–9.2
High decreasing	30 (0.9) ^a^	89 (2.8) ^b^	23 (2.3)	mean (SD)	18.6 (3.5)	17.6 (3.5)	14.6 (3.5)	14.7 (2.7)	13.4 (3.2)	13.9 (3.1)	10.5 (2.6)	10.5 (2.5)
				95% CI	17.8–19.3	16.1–19.1	13.9–15.4	13.6–15.7	12.6–14.1	12.6–15.2	9.6–11.3	9.4–11.6
Moderate increasing	76 (2.4) ^a^	169 (5.3) ^b^	67 (6.7)	mean (SD)	12.6 (2.9)	12.2 (3.0)	14.2 (3.4)	13.7 (3.6)	17.2 (3.4)	16.6 (3.7)	17.7 (2.9)	17.6 (2.7)
				95% CI	12.1–13.0	11.5–13.0	13.7–14.8	12.9–14.6	16.6–17.7	15.7–16.7	17.1–17.7	16.9–18.3
Stable high	99 (3.1) ^a^	360 (11.2) ^b^	82 (8.2)	mean (SD)	19.9 (3.0)	19.4 (3.0)	19.9 (3.1)	19.8 (3.2)	20.4 (2.9)	20.3 (3.0)	19.6 (3.7)	19.6 (3.4)
				95% CI	19.6–20.2	18.8–20.1	19.6–20.3	19.1–20.5	20.0–20.8	19.7–21.0	18.9–20.2	18.8–20.5
Fathers	*n* = 2068		*n* = 513									
Stable low	457 (14.3) ^a^	1657 (80.1) ^b^	425 (79.5)	mean (SD)	7.6 (2.1)	7.4 (2.1)	7.6 (2.3)	7.5 (2.2)	7.8 (2.4)	7.7 (2.4)	7.7 (2.4)	7.6 (2.4)
				95% CI	7.5–7.7	7.2–7.6	5.6–9.6	7.3–7.7	7.6–7.9	7.5–7.9	7.5–7.9	7.4–7.9
High decreasing	7 (0.2) ^a^	33 (1.6) ^b^	4 (0.3)	mean (SD)	19.1 (2.7)	19.7 (4.5)	13.7 (6.0)	11.8 (7.3)	12.6 (5.6)	12.3 (8.4)	9.5 (2.8)	8.3 (3.4)
				95% CI	18.2–20.0	14.6–24.8	11.3–16.0	4.6–18.8	10.1–15.2	2.8–21.2	7.5–11.5	4.9–11.6
Stable moderate	65 (2.0) ^a^	228 (11.0) ^b^	51 (12.5)	mean (SD)	14.4 (1.8)	14.6 (2.2)	13.5 (3.1)	13.4 (2.8)	13.4 (2.6)	13.2 (2.5)	12.5 (3.1)	12.7 (3.2)
				95% CI	14.2–14.6	14.0–15.2	13.0–14.0	12.6–14.1	12.9–13.8	12.5–13.9	11.8–13.2	11.8–13.7
Moderate increasing	20 (0.6) ^a^	80 (3.9) ^b^	23 (5)	mean (SD)	11.1 (2.4)	10.3 (1.4)	14.1 (3.1)	13.7 (2.6)	16.1 (2.5)	16.0 (2.3)	16.2 (2.5)	15.9 (2.1)
				95% CI	10.6–11.6	9.7–10.9	13.4–14.8	12.6–14.8	15.5–16.7	15.0–16.9	15.2–17.1	14.8–16.9
Stable high	10 (0.3) ^a^	70 (3.4) ^b^	10 (2.6)	mean (SD)	21.7 (2.3)	21.3 (2.4)	21.6 (2.6)	21.4 (2.0)	22.7 (2.4)	21.9 (2.4)	22.4 (2.0)	21.7 (2.3)
				95% CI	21.2–22.3	19.8–22.8	20.8–22.4	20.2–22.6	21.8–23.5	20.4–23.4	21.4–23.4	20.0–23.4

**Table 2:** Distribution of the participants according to their oral health-related quality of life (OHIP-14), age and education at gw34 and 4 yr time points.

		Mothers	Fathers
		*n* = 998	*n* = 513
Age	Mean (SD)	31.1 (4.28)	33.1 (3.51)
Education	Low, *n* (%)	284 (28.5)	197 (38.5)
	Medium, *n* (%)	279 (27.9)	160 (31.1)
	High, *n* (%)	435 (43.6)	156 (30.4)
OHIP-14 gw34	Severity score, mean (SD)	1.50 (3.2)	1.98 (4.2)
OHIP-14 4 yr	Severity score, mean (SD)	2.20 (4.0)	2.23 (4.1)
Change in OHIP-14	Severity score, mean (SD)	−0.71 (4.4)	−0.24 (4.5)
OHIP-14 dimensions gw34	Functional limitation, mean (SD)	0.08 (0.4)	0.18 (0.6)
	Physical pain	0.59 (1.1)	0.76 (1.3)
	Psychological discomfort	0.39 (0.9)	0.46 (1.1)
	Physical disability	0.07 (0.5)	0.10 (0.5)
	Psychological disability	0.21 (0.7)	0.23 (0.7)
	Social disability	0.07 (0.4)	0.11 (0.5)
	Handicap	0.09 (0.5)	0.14 (0.6)
OHIP-14 dimensions 4 yr	Functional limitation, mean (SD)	0.70 (0.4)	0.14 (0.6)
	Physical pain	0.84 (1.4)	0.87 (1.3)
	Psychological discomfort	0.63 (1.3)	0.59 (1.2)
	Physical disability	0.09 (0.5)	0.08 (0.4)
	Psychological disability	0.33 (0.9)	0.26 (0.8)
	Social disability	0.07 (0.4)	0.12 (0.5)
	Handicap	0.17 (0.6)	0.17 (0.6)

OHIP-14 = Oral Health Impact Profile 14; gw34 = gestational week 34; 4 yr = 4-year time point.

**Table 3:** Oral health-related quality of life (OHIP-14 severity) at two time points and the change in it according to dental anxiety trajectories.

Trajectories	*n* (%)	Severity Mean (SD)				
		gw34	4 Yr	Change	*p*-Value *	rc ***
Mothers	998	1.50 (3.3)	2.20 (4.0)	−0.71 (4.4)	<0.001	0.310
Stable low	826 (82.8)	1.33 (3.1)	1.88 (3.6)	−0.55 (4.1)	<0.001	0.273
High decreasing	23 (2.3)	1.35 (2.2)	2.48 (3.2)	−1.13 (3.9)	0.254	0.314
Moderate increasing	67 (6.7)	2.46 (3.6)	3.91 (4.5)	−1.45 (4.6)	0.006	0.446
Stable high	82 (8.2)	2.38 (4.9)	3.95 (6.5)	−1.57 (6.0)	0.007	0.428
*p*-value				0.006 **		
Fathers	513	1.98 (4.2)	2.23 (4.1)	−0.24 (4.5)	0.005	0.186
Stable low	425 (79.5)	1.75 (3.7)	1.87 (3.4)	−0.12 (4.3)	0.067	0.014
High decreasing	4 (0.3)	5.50 (8.0)	2.00 (2.4)	3.50 (7.0)	0.317	n.a.
Stable moderate	51 (12.5)	3.33 (6.9)	4.12 (6.2)	−0.78 (5.7)	0.112	0.294
Moderate increasing	23 (5)	1.83 (3.3)	2.39 (2.5)	−0.57 (3.3)	0.220	0.358
Stable high	10 (2.6)	4.10 (5.7)	7.60 (8.7)	−3.50 (4.6)	0.030	0.861
*p*-value				0.403 **		

OHIP-14 = Oral Health Impact Profile 14; gw34 = gestational week 34; 4 yr = 4-year time point; * Wilcoxon signed-rank test, difference between time points; ** Jonckheere–Terpstra test, difference in change between dental anxiety trajectory classes; *** matched-pairs rank biserial correlation coefficient.

**Table 4:** The mean change in oral health-related quality of life (OHIP-14) dimensions according to dental anxiety trajectories.

		Change in the Mean (SD)						
Dental Anxiety Trajectories	*n* (%)	Functional Limitation	Physical Pain	Psychological Discomfort	Physical Disability	Psychological Disability	Social Disability	Handicap
Mothers	998 (100)	0.01 (0.5)	−0.25 (1.6)	−0.24 (1.3)	−0.02 (0.6)	−0.12 (1.0)	−0.00 (0.6)	−0.08 (0.7)
Stable low	826 (82.8)	0.01 (0.5)	−0.22 (1.5)	−0.20 (1.2)	−0.02 (0.6)	−0.10 (0.2)	0.01 (0.6)	−0.04 (0.6)
High decreasing	23 (2.3)	−0.04 (0.2)	−0.43 (2.1)	−0.26 (1.3)	0.13 (0.7)	−0.04 (0.6)	−0.13 (0.7)	−0.35 (0.8)
Moderate increasing	67 (6.7)	0.03 (0.4)	−0.37 (1.9)	−0.55 (1.5)	0.06 (0.7)	−0.33 (1.3)	−0.06 (0.6)	−0.22 (0.8)
Stable high	82 (8.2)	−0.07 (0.9)	−0.48 (1.9)	−0.41 (2.0)	−0.06 (0.6)	−1.59 (0.04)	−0.64 (0.1)	−0.83 (0.3)
*p*-value		0.637	0.131	0.034	0.104	0.046	0.078	<0.001
Fathers	513 (100)	0.04 (0.7)	−0.11 (1.5)	−0.13 (1.3)	0.02 (0.6)	−0.03 (0.8)	−0.006 (0.7)	−0.03 (0.7)
Stable low	425 (82.8)	0.04 (0.6)	−0.08 (1.5)	−0.07 (1.2)	0.01 (0.6)	−0.03 (0.8)	0.005 (0.6)	0.01 (0.6)
High decreasing	4 (0.8)	0.00 (0.8)	1.25 (1.9)	0.50 (1.7)	0.50 (1.0)	0.50 (1.7)	0.75 (2.2)	0.00 (0.8)
Stable moderate	51 (9.9)	0.10 (0.9)	−0.23 (1.7)	−0.41 (1.3)	0.12 (0.7)	−0.14 (0.8)	0.04 (0.8)	−0.25 (0.8)
Moderate increasing	23 (4.5)	0.00 (0.7)	−0.35 (1.5)	−0.48 (1.3)	0.09 (0.4)	0.22 (0.6)	0.04 (0.4)	−0.09 (0.6)
Stable high	10 (1.9)	−0.10 (0.7)	−0.80 (1.6)	−0.70 (1.5)	−0.40 (1.0)	−0.60 (0.7)	−0.70 (1.1)	−0.20 (0.6)
*p*-value		0.523	0.965	0.126	0.804	0.005	0.346	0.019

*p*-values for Jonckheere–Terpstra test.

## Data Availability

Data are not available due to restrictions related to privacy and ethical issues. The datasets presented in this article are not readily available because of restrictions based on EU GDPR and local legislation. Requests to access the datasets should be directed to L.K.
